# Simvastatin causes pulmonary artery relaxation by blocking smooth muscle ROCK and calcium channels: Evidence for an endothelium-independent mechanism

**DOI:** 10.1371/journal.pone.0220473

**Published:** 2019-08-01

**Authors:** Mais Absi, Basma G. Eid, Nick Ashton, George Hart, Alison M. Gurney

**Affiliations:** 1 Faculty of Biology Medicine and Health, University of Manchester, Core Technology Facility level, Manchester, United Kingdom; 2 Cardiovascular Sciences, Kings College London, London, United Kingdom; 3 Department of Pharmacology and Toxicology, Faculty of Pharmacy, King Abdulaziz University, Jeddah, Saudi Arabia; Indiana University School of Medicine, UNITED STATES

## Abstract

Simvastatin reduces pulmonary arterial pressure and right ventricular hypertrophy in animal models of pulmonary arterial hypertension (PAH) and is thought to restore endothelial dysfunction. *In vivo* effects of drugs are complicated by several factors and little is known of the direct effects of statins on pulmonary arteries. This study investigated the direct effects of simvastatin on pulmonary arteries isolated from rats with or without monocrotaline-induced PAH. Simvastatin suppressed contractions evoked by the thromboxane A2 receptor agonist U46619 (30 nM), the α_1_–adrenergic agonist phenylephrine (5 μM) and KCl (50 mM) by ~50% in healthy and diseased arteries, but did not reduce contraction evoked by sarco/endoplasmic reticulum ATPase blockers. It relaxed hypertensive arteries in the absence of stimulation. Removing the endothelium or inhibiting eNOS did not prevent the inhibition by simvastatin. Inhibiting RhoA/rho kinase (ROCK) with Y27632 (10 μM) suppressed contractions to U46619 and phenylephrine by ~80% and prevented their inhibition by simvastatin. Y27632 reduced KCl-induced contraction by ~30%, but did not prevent simvastatin inhibition. Simvastatin suppressed Ca^2+^ entry into smooth muscle cells, as detected by Mn^2+^ quench of fura-2 fluorescence. The calcium antagonist, nifedipine (1 μM), almost abolished K^+^-induced contraction with less effect against U46619 and phenylephrine. We conclude that simvastatin relaxes pulmonary arteries by acting on smooth muscle to interfere with signalling through G-protein coupled receptors and voltage-dependent Ca^2+^ entry. Its actions likely include inhibition of ROCK-dependent Ca^2+^ sensitisation and voltage-gated Ca^2+^ channels. These are likely to contribute to the beneficial effects of simvastatin in animal models of PAH.

## Introduction

Statins have protective effects on the cardiovascular system independently of their cholesterol-lowering action [1]. An oral dose of serivastatin enhanced systemic, endothelium-dependent vasodilation in patients with normal serum cholesterol levels within 3h [2], the time required to reach peak plasma concentrations [3]. Endothelial nitric oxide synthase (eNOS) activity increased within 30 min of statin exposure *in vitro* [4]. Such rapid effects likely involve post-translational activation of the eNOS protein. By inhibiting HMG-CoA reductase, statins prevent the synthesis of mevalonate and downstream isoprenoid intermediates required for activation of the RhoA Rho kinase (ROCK) signalling pathway [1]. In endothelial cells, ROCK is a negative regulator of the protein kinase Akt, which phosphorylates and activates eNOS [5]. Statins can therefore enhance eNOS activity by removing RhoA/ROCK inhibition of Akt.

Statins also inhibit RhoA/ROCK activity in vascular smooth muscle [1,6–8], where it plays a key role in sensitising the contractile machinery to Ca^2+^ and promoting contraction [9]. Consequently ROCK inhibitors suppress the effects of vasoconstrictors [10,11]. Additional actions of simvastatin could contribute to its vasodilator effect. For example, it was found to inhibit Ca^2+^ channels in rat basilar artery [12] and cardiac [13] myocytes and to interfere with sarcoplasmic reticulum (SR) Ca^2+^ handling in aortic muscle [14]. This may be why simvastatin suppressed Ca^2+^ signalling in basilar arteries [12] and cultured arterial smooth muscle cells [8,15]. Simvastatin was alternatively proposed to suppress mesenteric artery constriction by stimulating AMP-activated protein kinase (AMPK) to phosphorylate eNOS and enhance constitutive eNOS activity [16].

Less is known about the effects of statins on the pulmonary circulation, although they attenuate pulmonary arterial hypertension (PAH) in animal models. Simvastatin reduced pulmonary arterial pressure and right ventricular hypertrophy in rats with PAH, caused by monocrotaline (MCT) injection [17] or chronic exposure to hypoxia with or without blockade of the vascular endothelial growth factor receptor [18–20]. The beneficial effects were attributed to improved endothelial function, reduced inflammation and reduced smooth muscle cell proliferation. Restoration of endothelium-dependent relaxation may be due to stabilization of eNOS mRNA and increased eNOS protein [7,20,21]. Our knowledge of statin effects on the pulmonary circulation has mainly come from *in vivo* studies with chronic statin treatment, where they are influenced by indirect actions on the heart, nervous system or other organs, as well as compensatory changes in the vasculature or drug metabolites. This study aimed to determine the direct effects of simvastatin applied acutely to pulmonary artery (PA) and to assess the relative contributions of endothelial and smooth muscle actions. The monocrotaline rat model of pulmonary hypertension was employed to determine how disease status affects simvastatin action.

## Materials and methods

Animal studies are reported according to ARRIVE and BJP guidelines [22]. Work was conducted with the authority of a licence granted under the UK Animals (Scientific Procedures) Act 1986 and adhered to the guidelines of Directive 2010/63/EU of the European Parliament on the protection of animals used for scientific purposes.

### Monocrotaline model of pulmonary arterial hypertension

As our understanding of statin effects in PAH is derived mainly from studies on the rat monocrotaline model, we used this model to assess how disease-associated changes in artery function influence simvastatin action. Animals were maintained in controlled temperature and light conditions with free access to food and water. Male Wistar rats (Charles River, UK) randomly received a single intraperitoneal injection of MCT (60 mg. kg^-1^) or an equivalent volume of 0.9% saline and were weighed and observed daily. On the day of injection, the control (saline-injected) and MCT rats weighed 236 ± 3 g (n = 30) and 235 ± 3 g (n = 30), respectively. Animals were sacrificed by cervical dislocation four weeks after MCT injection or earlier if they showed signs of clinical deterioration, such as reduced movement, increased respiratory rate, piloerection, or >10 g weight loss over 2 days. At termination, the MCT rats had gained less weight (305 ± 4 g) than the time-matched controls (367 ± 5 g). Lungs and heart were excised into physiological salt solution (PSS) containing (in mM): 120 NaCl, 5 KCl, 1 MgCl_2_, 0.5 NaH_2_PO_4_, 0.5 KH_2_PO_4_, 10 4-(2-hydroxyethyl)piperazine-1-ethanesulphonic acid (HEPES), 5 glucose and 1.8 CaCl_2;_ pH adjusted to 7.4 with NaOH. The ileum with attached mesentery was removed and stored separately in PSS. The right ventricle (RV) was separated from the left ventricle plus interventricular septum (LV+S) and each weighed separately. The development of RV hypertrophy, and hence PAH, was confirmed by a higher RV/(LV+septum) weight ratio in the MCT rats (0.6 ± 0.01) compared with the controls (0.4 ± 0.02).

**Fig 1:**
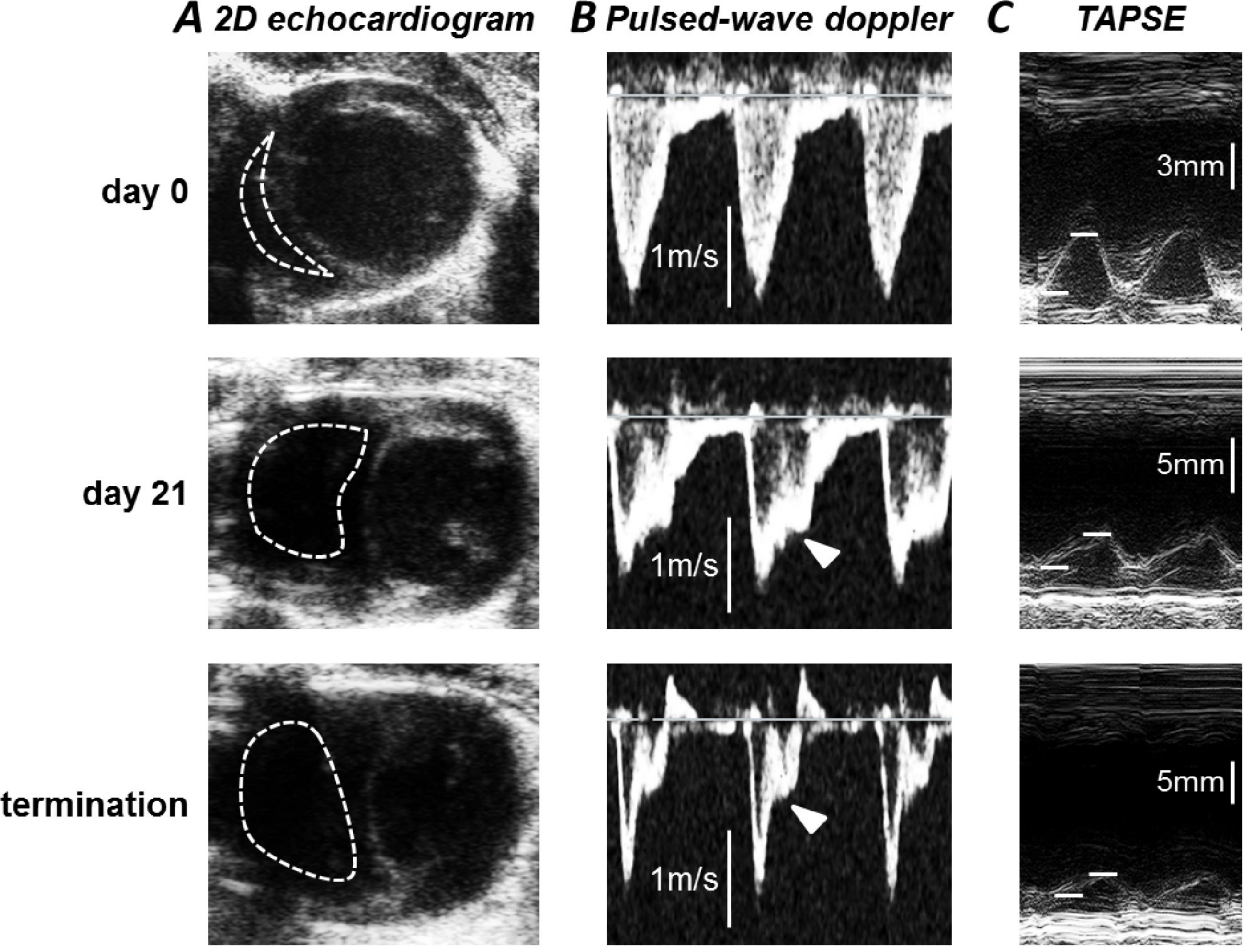
Confirmation of PAH by echocardiography. Echocardiographic images collected from rats immediately before injection (day 0), at 21 days after injection and immediately prior to termination. ***A***) Parasternal short-axis views with right ventricle outlined by dotted lines. ***B***) Pulsed-wave Doppler recordings of pulmonary outflow. Arrowheads indicate notching in the images from treated rats. ***C***) M-mode imaging of the lateral tricuspid annulus. TAPSE was measured as the total displacement of the annulus from end-diastole (marked by lower line) to end-systole (upper line).

Disease progression was monitored by echocardiography performed under 2% isoflurane anaesthesia: parameters confirming the development of PAH are summarised in Fig 1 and Table 1. Transthoracic echocardiographic images were acquired on an ACUSON Sequoia system (Acuson Universal Diagnostics Solution, USA) with a 15 MHz 15L8 transducer. Two-dimensional images were generated in B-mode from the parasternal short axis view and used to measure right (RV) and left (LV) ventricle areas at the end of diastole. RV fractional shortening was measured from the difference in diameter of the right ventricle outflow tract between end-diastole and end-systole. Pulsed-wave Doppler was used to measure PA acceleration time (PAAT) and trans-mitral flow velocity. The latter parameter was measured as the ratio of the peak velocity in early diastole (E) to the end diastolic flow velocity (A). RV stroke volume and output were calculated from the PA flow velocity time integral, measured from pulsed-wave Doppler traces. Tricuspid annular plane systolic excursion (TAPSE) was measured in M-mode from two-dimensional images of the lateral tricuspid annulus, as the total displacement of the tricuspid annulus from end-diastole to end-systole.

**Table 1:** Echocardiographic Indicators of cardiac function and pulmonary arterial blood flow.

Property	control	MCT Day 21	MCT Day 25
Heart Rate (beat per minute)	379 ± 4 (6)	354 ± 7 (9)	338 ± 11* (7)
RV/LV area ratio	0.4 ± 0.04 (6)	1.6 ± 0.2* (9)	1.3 ± 0.2* (8)
RV fractional shortening (%)	44 ± 3 (5)	41 ± 3 (7)	35 ± 7 (7)
PA acceleration time (ms)	31 ± 1 (12)	21 ± 2* (9)	20 ± 2* (8)
Tricuspid valve flow velocity (E/A ratio)	0.7± 0.1 (6)	0.5 ± 0.1 (6)	0.6 ± 0.1 (5)
RV stroke volume (ml)	0.53 ± 0.1 (6)	0.45 ± 0.03 (9)	0.38 ± 0.04 (8)
RV output (ml/min)	199 ± 24 (6)	161 ± 13 (9)	130 ± 15* (8)
TAPSE (mm)	2.6 ± 0.1 (6)	1.9 ± 0.15* (9)	1.8 ± 0.07* (8)

Measurements were made immediately before injection with MCT (control) and at 21 and 25 days after injection. Numbers of animals are indicated in parentheses.

*differs significantly from control, one-way ANOVA with Tukey’s multiple comparisons test.

At day 21 after MCT injection there was an increase in right ventricle cross-sectional area at diastole, associated with a reduced cross-sectional area of the left ventricle (Fig 1A), presumably due to the raised right ventricular pressure pushing the septum into the left ventricle. The RV/LV ratio was therefore increased (Table 1) in agreement with the increased RV/(LV+septum) weight ratio. Despite these changes, cardiac function appeared to be maintained at day 21, as indicated by heart rate, trans-mitral flow velocity, RV fractional shortening, stroke volume and output (Table 1). Impaired RV function was apparent in these markers at day 25, although the reduction in TAPSE (Table 1) indicates some loss of function even at day 21. TAPSE correlates well with RV ejection fraction and provides a sensitive and reliable marker of RV function [23].

A decrease in PAAT and the appearance of a sharp peak, or notch, in the early systolic phase of the PA flow waveform (Fig 1B) are established features of PAH [24, 25] and were detected by day 21 (Fig 1B, Table 1). A strong correlation between PAAT and mean pulmonary arterial pressure (mPAP) means that mPAP can be determined from the following equation: mPAP = 58.7 –(1.21 *x* PAAT) [24]. From the values of PAAT in Table 1, the mPAPs of the rats employed in this study were estimated at 21 mmHg in control rats, increasing to 33 mmHg and 34.5 mmHg at 21 and 25 days following MCT injection, respectively.

The data confirm the presence of PAH by 21 days after MCT injection. Further characterisation of PAH development in MCT-injected rats in our hands is published elsewhere [26].

### Myograph protocols

Segments of pulmonary or mesenteric artery (~2 mm long, 0.5–1 mm outer diameter) were dissected from lung and mesentery, respectively, and mounted in a small vessel myograph (Danish Myo Technology, Aarhus, Denmark) under 4–5 mN tension, in PSS at 37°C and bubbled with air. To minimise animal use, multiple vessel segments from the 3 largest lung lobes were used for different experiments performed simultaneously. Vessels were left for >20 min to recover from dissection and stability checked by challenging three times with 50 mM KCl. Simvastatin (5 μM) was added to some chambers for 1 hr before experiments started and remained throughout the experiment. Vessel constriction was induced with 50 mM KCl, the α_1_-adrenoceptor agonist phenylephrine (5 μM), the thromboxane A2 (TP) receptor agonist 9,11-dideoxy-9a,11a-methanoepoxy prostaglandin F_2_α (U46619, 30 nM) or inhibitors of the sarco-endoplasmic reticulum ATPase (SERCA), thapsigargin (3 μM) and cyclopiazonic acid (CPA, 30 μM). To investigate AMPK involvement in the effects of simvastatin, the cell-permeable inhibitor dorsomorphin (also known as compound C) was applied at 1 μM, a 10-fold higher concentration than needed for 50% reduction of AMPK activity (109 nM) [27]. ROCK activity was inhibited with Y27632 (*trans*-4-[(1*R*)-1-aminoethyl]-N-4-pyridinylcyclohexanecarboxamide dihydrochloride) at 10 μM, a maximally effective concentration for ROCK inhibition and relaxation of rat PA and aorta [11]. The calcium antagonist nifedipine was employed at a concentration (1 μM) shown to maximally inhibit L-type Ca^2+^ channels in PA smooth muscle cells [28].

Acetylcholine (ACh; 10^−8^–10^−5^ M) was applied to evoke endothelium-dependent relaxation and sodium nitroprusside (SNP; 10^−9^–10^−6^ M) to deliver NO directly to smooth muscle: drugs were applied to vessels constricted with 30 nM U46619 once tension reached a plateau. In some experiments, endothelial function was blocked by passing distilled water through the vessel lumen or applying N(G)-nitro-L-arginine methyl ester (L-NAME, 200 μM) to inhibit eNOS activity. Loss of function was confirmed by the absence of vasodilation to 10 μM ACh. Experiments employed intact arteries unless otherwise indicated.

### Rho kinase activity

All arteries from all lung lobes were collected to provide sufficient protein for the measurement of ROCK activity. Before dissecting the lungs, a 0.5 ml bubble of air was injected via the main pulmonary artery to disrupt the endothelium, and washed through with PSS, although its effectiveness could not be checked in all vessels. Arteries were divided into four groups and incubated for 1 hour in 5 μM simvastatin or an equivalent volume of vehicle, with or without the addition of 30 nM U46619, then snap frozen in liquid nitrogen. Tissue lysates were prepared by homogenising arteries in solution containing 150 mM NaCl, 15 mM HEPES, 10 mM EGTA (pH 7.5 with NaOH), cOmplete protease inhibitor cocktail (Roche, 1 tablet/ 30 ml) and PhosSTOP phosphatase inhibitor cocktail (Roche, I tablet/10ml). Homogenates were centrifuged at 16,000g for 5 min at 5°C and the supernatant collected for analysis. ROCK activity was determined using an immunoassay kit that measured phosphorylation of the Rho kinase-specific substrate, MYPT-1 (Merck), according to the manufacturer’s instructions. Lysate activity was measured by comparison to a ROCK2 standard provided in the kit and expressed relative to total protein, measured using the Bio-Rad *DC* Protein Assay.

### Ca^2+^ influx

Pulmonary artery smooth muscle cells were isolated as previously described [29] then incubated for 30 min at room temperature with the Ca^2+^-sensitive fluorescent indicator fura-2 AM (2 μM; Molecular Probes, Sunnyvale, CA). Fluorescence was excited in individual cells by illuminating alternatively at 340 nm and 380 nm (100Hz, 75W Xenon arc lamp) using a monochromator-based fluorescence system (Optiscan, Cairn Research, Kent, UK), integrated with a Nikon Eclipse inverted microscope. The fluorescence emitted at 510 nm was recorded using a photomultiplier tube (Cairn Research) and pClamp software (Axon Instruments, Union City, CA). Cells were incubated for 1 hour with vehicle or 5 μM simvastatin (during and after fura-2 loading) before experiments began. To evoke depolarisation and Ca^2+^ channel activation without Ca^2+^ influx, cells were incubated with PSS modified to contain 50 mM K^+^ and no added Ca^2+^. Osmolarity was maintained by equimolar substitution of Na^+^. These conditions evoke smooth muscle depolarisation to open Ca^2+^ channels while minimising Ca^2+^-dependent fluorescence. Cell fluorescence was recorded for 1 min before adding 1 mM MnCl_2_, allowing Mn^2+^ to enter the cell and quench fura-2 fluorescence [30]. The rate of Mn^2+^ quenching was measured from the fluorescence excited at 380 nm.

### Data and statistical analysis

The data and statistical analyses comply with the recommendations on experimental design and analysis in pharmacology [31]. Contraction amplitude was measured as the difference between baseline and induced tension. Relaxation was measured as percent of the maintained U46619-induced contraction. The maximum effect (E_max_) and drug concentration producing half of the E_max_ (EC_50_) were estimated from individual concentration–response relationships. Mean concentration-response relationships are plotted with nonlinear regression fits of the Hill equation (GraphPad Prism, version 6) superimposed. Values are expressed as mean ± standard error of mean (S.E.M.) for the number of animals (n) used in each experiment. Data were tested for normality using the Shapiro-Wilk and Kolmogorov-Smirnov tests and comparisons made with parametric or non-parametric tests as appropriate. Significance was corrected for multiple comparisons using the test recommended by Prism for each data set. Tests used are indicated next to the comparisons made. P<0.05 was considered statistically significant.

### Drugs

U46619 was obtained from Enzo Life Sciences (Exeter, UK). All other drugs and reagents were from Sigma-Aldrich (Dorset, UK).

## Results

### PAH and simvastatin inhibit contraction

**Fig 2:**
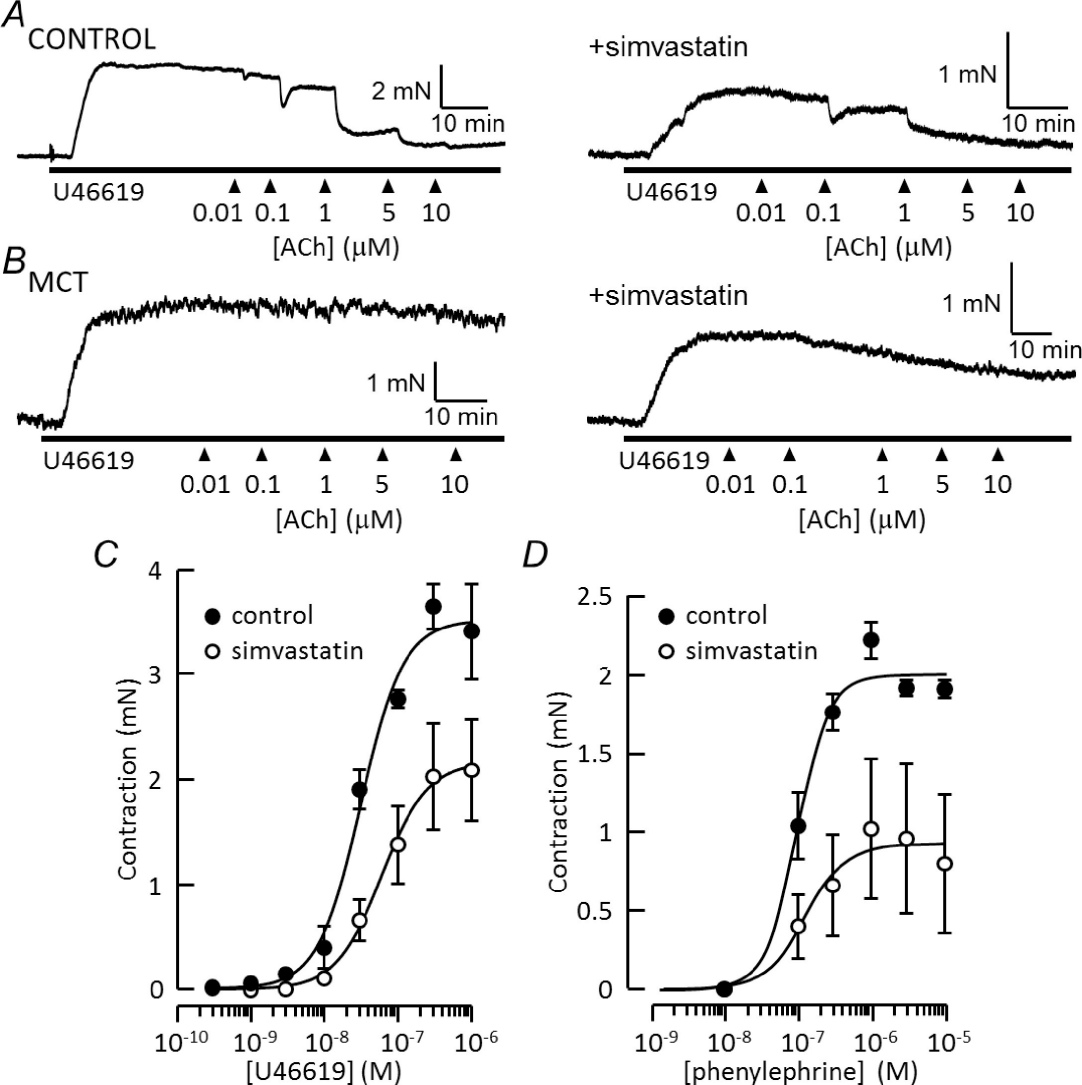
Simvastatin inhibits pulmonary artery reactivity. ***A*, *B***) Original records of tension developed by pulmonary arteries in response to 30 nM U46619, followed by increasing concentrations of ACh (10 nM– 10 μM). Records from paired vessels treated with vehicle (left) or 5 μM simvastatin (right) from control (*A*) and MCT-treated (*B*) rats. ***C*,*D***) Concentration response curves for U46619 and phenylephrine acting on control vessels, under control conditions and after incubation for 1 hour with 5 μM simvastatin. Points and bars represent mean ± s.e.m of vessels from 4 animals.

**Fig 3:**
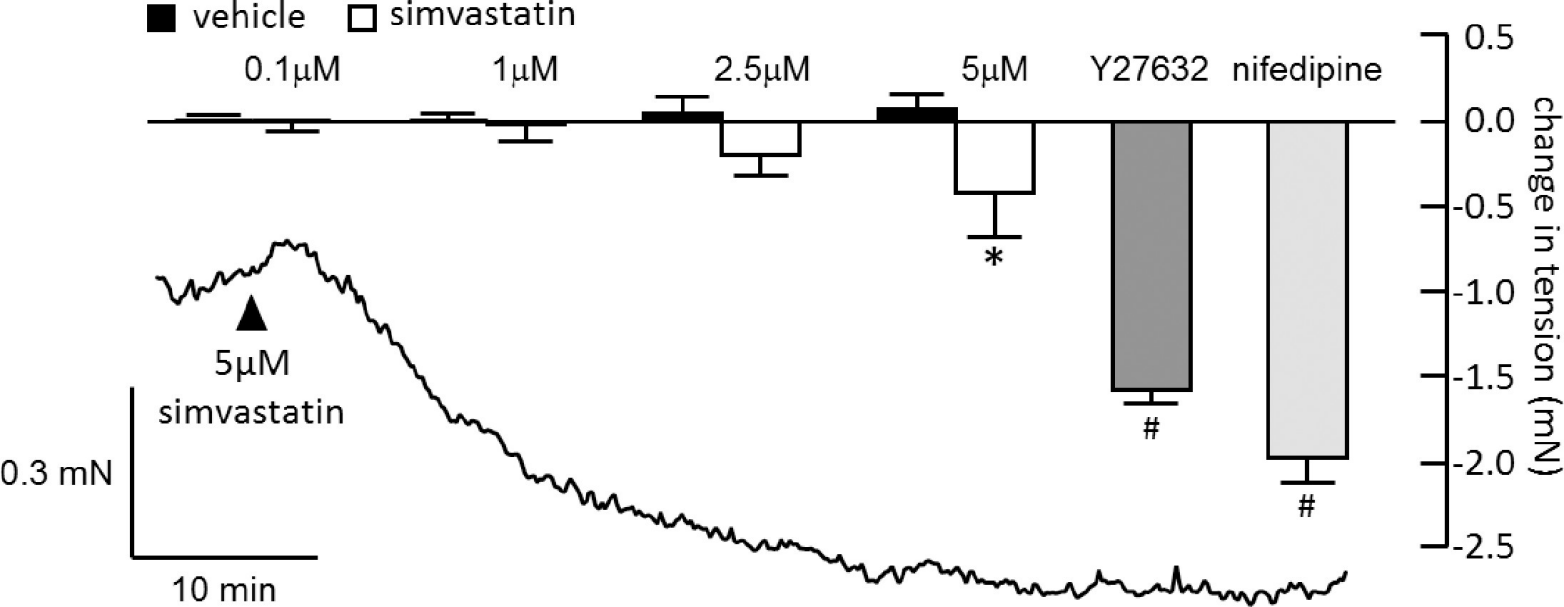
Effects of simvastatin, ROCK and calcium-channel block on basal tone of diseased arteries. Trace is original record of baseline tension before and during the application of 5 μM simvastatin. Histogram shows the mean change in tension following application of simvastatin (0.1–5 μM) or vehicle (n = 4), 10 μM Y27632 (n = 10) or 1 μM nifedipine (n = 5) to arteries from MCT rats at resting tension. *differs significantly from time-matched control (2-way repeated measures (RM) ANOVA and Sidak multiple comparisons test of each simvastatin concentration versus the vehicle control); ^#^differs significantly from 5 μM simvastatin (one-way ANOVA with Tukey multiple comparisons test, each group compared with every other group).

PAs from control and MCT-treated rats contracted upon application of U46619 (30 μM), in the absence or presence of 5 μM simvastatin (Fig 2*A* and 2*B*). Contraction amplitude was similar in control and MCT vessels but halved by simvastatin (Table 2). Contractions to phenylephrine (5 μM) and KCl (50 mM) were reduced in MCT vessels compared with the controls, but in both cases contraction was ~50% smaller after simvastatin treatment (Table 2). In contrast, contractions of mesenteric artery to KCl and phenylephrine were unaltered in rats with PAH (Table 2). Fig 2C and 2D show that simvastatin reduced the maximum contractile responses to U46619 and phenylephrine with little effect on sensitivity. U46619 evoked up to 3.7 ± 0.2 mN before simvastatin treatment and 2.2 ± 0.5 mN (n = 4, p<0.05 by unpaired t-test) after treatment, with respective EC_50_ values of 31 ± 5 nM and 61 ± 20 nM. The maximum contraction to phenylephrine dropped from 2.2 ± 0.1 mN (control) to 1.0 ± 0.4 mN (n = 4, p<0.05) after simvastatin treatment, with EC_50_ values of 112 ± 8 nM and 187 ± 59 nM, respectively. Simvastatin (5 μM) had no effect on baseline tension when applied to control vessels, but in vessels from MCT rats it produced a concentration-dependent loss of tone (Fig 3).

**Table 2:** PAH and simvastatin inhibit pulmonary artery contraction.

			Contraction (mN)					
			KCl		U46619		phenylephrine	
artery	condition	simvastatin	mean ± s.e.m.	n	mean ± s.e.m.	n	mean ± s.e.m.	n
**pulmonary +E**	**control**	**-**	5.0 ± 0.5	23	3.9 ± 0.3	26	3.5 ± 0.4	20
		**+**	2.4 ± 0.4*	20	2.0 ± 0.2*	28	1.9 ± 0.3*	19
	**MCT**	**-**	2.7 ± 0.4^#^	18	3.4 ± 0.3	27	1.8 ± 0.4^#^	12
		**+**	0.8 ± 0.2*	17	1.1 ± 0.1*	30	1.1 ± 0.3	10
**pulmonary -E**	**control**	**-**	5.2 ± 0.3	9	3.3 ± 0.7	9	4.5 ± 0.5	9
		**+**	2.2 ± 0.3*	9	1.7 ± 0.4*	9	2.7 ± 0.4*	9
**Mesenteric**	**control**	**-**	6 ± 1.6	4			11 ± 2.8	4
	**MCT**	**-**	6 ± 2.3	4			11 ± 5	4

Contractile responses of pulmonary and mesenteric arteries from control and MCT-treated rats to 50 mM KCl, 30 nM U46619 and 5 μM phenylephrine, tested in the absence or presence of 5 μM simvastatin. Responses of intact (E+) pulmonary arteries to each stimulus, compared by two-way ANOVA and Tukey’s multiple comparisons test. Mesenteric and endothelium-denuded (E-) pulmonary arteries analysed by unpaired t-test.

*differ significantly from the equivalent data in the absence of simvastatin.

^#^differ significantly from the equivalent control data. n = number of animals.

Removing the endothelium did not prevent simvastatin from inhibiting KCl, U46619 or phenylephrine contractions, which were all roughly halved (Table 2). L-NAME (200 μM) also had no effect; simvastatin reduced the KCl response by 59 ± 10% (n = 5) in its presence and 60 ± 12% (n = 5) in its absence. Adding L-NAME raised baseline tone in control vessels by 1.4 ± 0.3 mN (n = 35) and in MCT vessels by 1.3 ± 0.2 mN (n = 33), but after simvastatin treatment L-NAME evoked contraction of only 0.20 ± 0.05 mN (n = 15) in control vessels (significantly less than control, one-way ANOVA with Tukey’s multiple comparisons test) and 0.9 ± 0.3 mN (n = 10) in MCT vessels. The inhibitory effect of simvastatin on vasoconstrictor responses was also unaffected by the AMPK inhibitor dorsomorphin (1 μM). U46619 evoked 0.9 ± 0.3 mN (n = 4) of tension in simvastatin-treated vessels before adding dorsomorphin and 0.9 ± 0.4 mN (n = 4) in its presence. KCl evoked 1.1 ± 0.3 mN (n = 4) in simvastatin-treated vessels before applying dorsomorphin and 1.3 ± 0.3 mN (n = 4) after its addition. Dorsomorphin had no effect in the absence of simvastatin.

**Fig 4:**
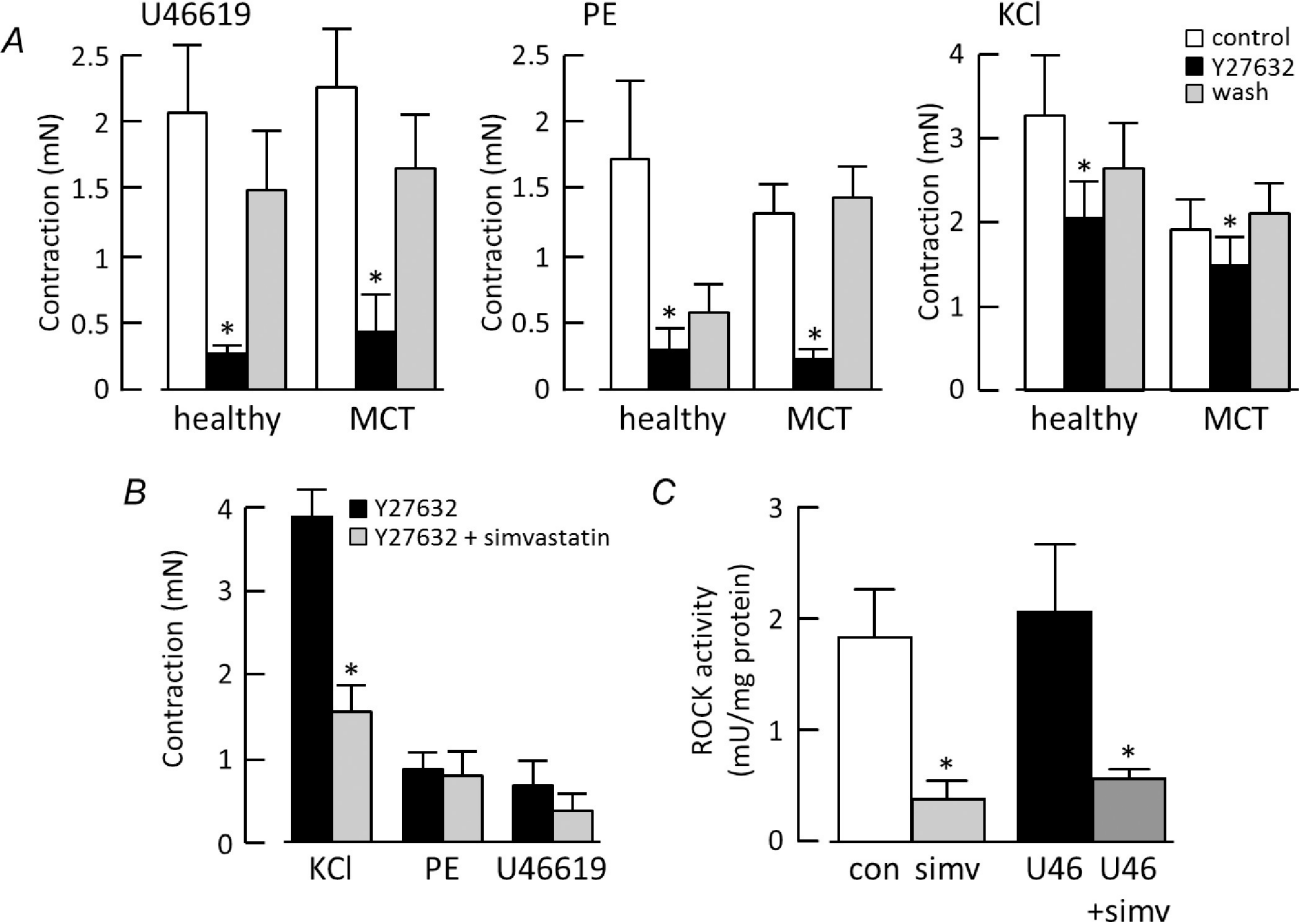
ROCK inhibition partly mimics simvastatin. ***A***) Contraction of healthy and MCT arteries to 30 nM U46619 (n = 6–7), 5 μM phenylephrine (PE, n = 6) and 50 mM KCl (n = 8) in control conditions, after 10 min exposure to 10 μM Y27632 and after 30 min of washing. * differs significantly from control by 2-way RM ANOVA with Tukey’s multiple comparisons test. ***B***) Comparison of healthy vessel contraction to KCl, phenylephrine and U46619 in the presence of 10 μM Y27632, before and after exposure to simvastatin. * differs significantly from KCl in Y27632 by paired t-test. ***C***) ROCK activity in arteries from 9 rats in the absence (con) or presence of 30 nM U46619 (U46), with or without the addition of 5 μM simvastatin (simv). *differs significantly from control and U46619 alone by one-way ANOVA with Tukey’s multiple comparisons test.

As shown in Fig 4A, the ROCK inhibitor Y27632 (10 μM) caused pronounced (~80%) inhibition of the contractile responses of control and MCT arteries to U46619 and phenylephrine. Y27632 also reduced the contractile response to KCl, but by only 33 ± 2% (n = 5) in control arteries and 21 ± 5% (n = 8) in MCT arteries. Although Y27632 inhibits endothelial and smooth muscle ROCK, removing the endothelium had little effect on the results (S1 Fig). Blocking ROCK activity with Y27632 prevented simvastatin (5 μM) from inhibiting contraction to phenylephrine and U46617, but not KCl (Fig 4*B*). U46619 did not evoke a detectable increase in ROCK activity, but activity was suppressed by 5 μM simvastatin whether U46619 was present or not (Fig 4C).

**Fig 5:**
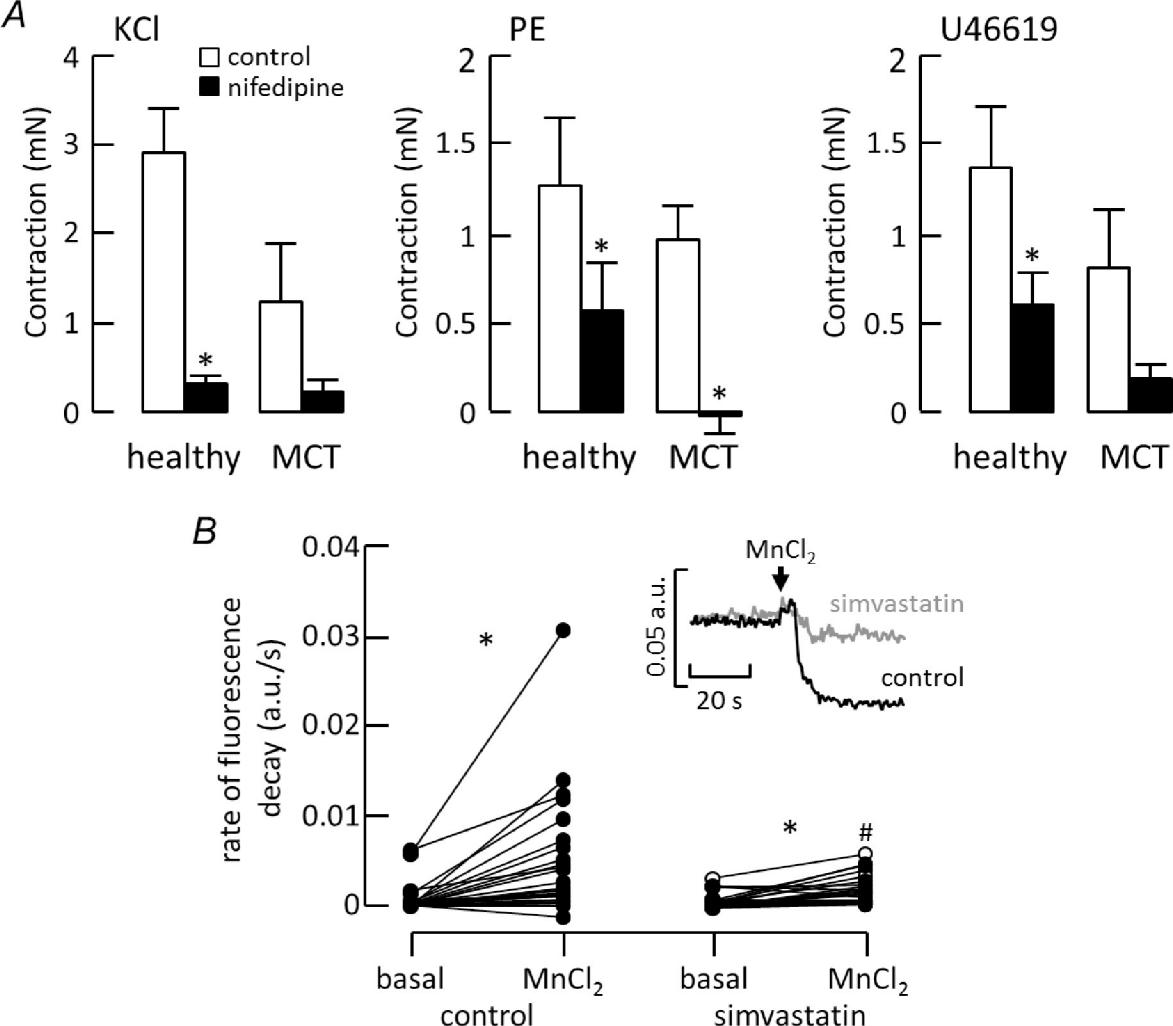
Simvastatin and Ca^2+^ influx. ***A***) Contraction of healthy and MCT arteries to KCl (healthy n = 7, MCT n = 3), phenylephrine (n = 6–7) and U46619 (n = 5) before (control) and after 10 min exposure to 1 μM nifedipine. *p<0.05 compared with matched control (2-way RM ANOVA with Sidak post-test). ***B***) Inset: fluorescence (380 nm) recordings from fura-2 loaded pulmonary artery smooth muscle cells in control conditions or the presence of simvastatin, before and after applying 1 mM MnCl_2_. Plot shows the increase in fluorescence decay upon exposure to MnCl_2_ in control conditions (n = 28 cells from 5 artery preparations) and after incubation with 5 μM simvastatin (n = 27 cells from 5 arteries). MnCl_2_ was applied at 1 mM (closed symbols) except for 7 simvastatin-treated cells exposed to 10 mM MnCl_2_ (open symbols). Wilcoxon matched pairs signed rank test indicates a significant difference between basal and MnCl_2_ in each condition* and between MnCl_2_ in the absence or presence of simvastatin^#^.

In contrast to the effects of Y27632, the calcium antagonist, nifedipine (1 μM), almost abolished the contraction to KCl (Fig 5A), confirming that it depended on voltage-gated Ca^2+^ influx. Nifedipine was less effective in control vessels stimulated by PE or U46619, where it caused around 50% inhibition, although its effects were enhanced in arteries from MCT rats (Fig 5*A*). This differential sensitivity to nifedipine reflects the relative contribution of L-type Ca^2+^ channels to contraction evoked by different stimuli and was essentially unchanged in vessels denuded of endothelium (S1 Fig). In separate studies on isolated pulmonary artery smooth muscle cells, simvastatin slowed the rate at which Mn^2+^ entered the cells and quenched fura-2 fluorescence (Fig 5B), implying that it inhibited Ca^2+^ influx. Neither Y7632 nor nifedipine altered basal tension in control arteries, but when added to arteries from MCT-treated rats, they both evoked relaxation that was larger than produced by 5 μM simvastatin (Fig 3).

**Fig 6:**
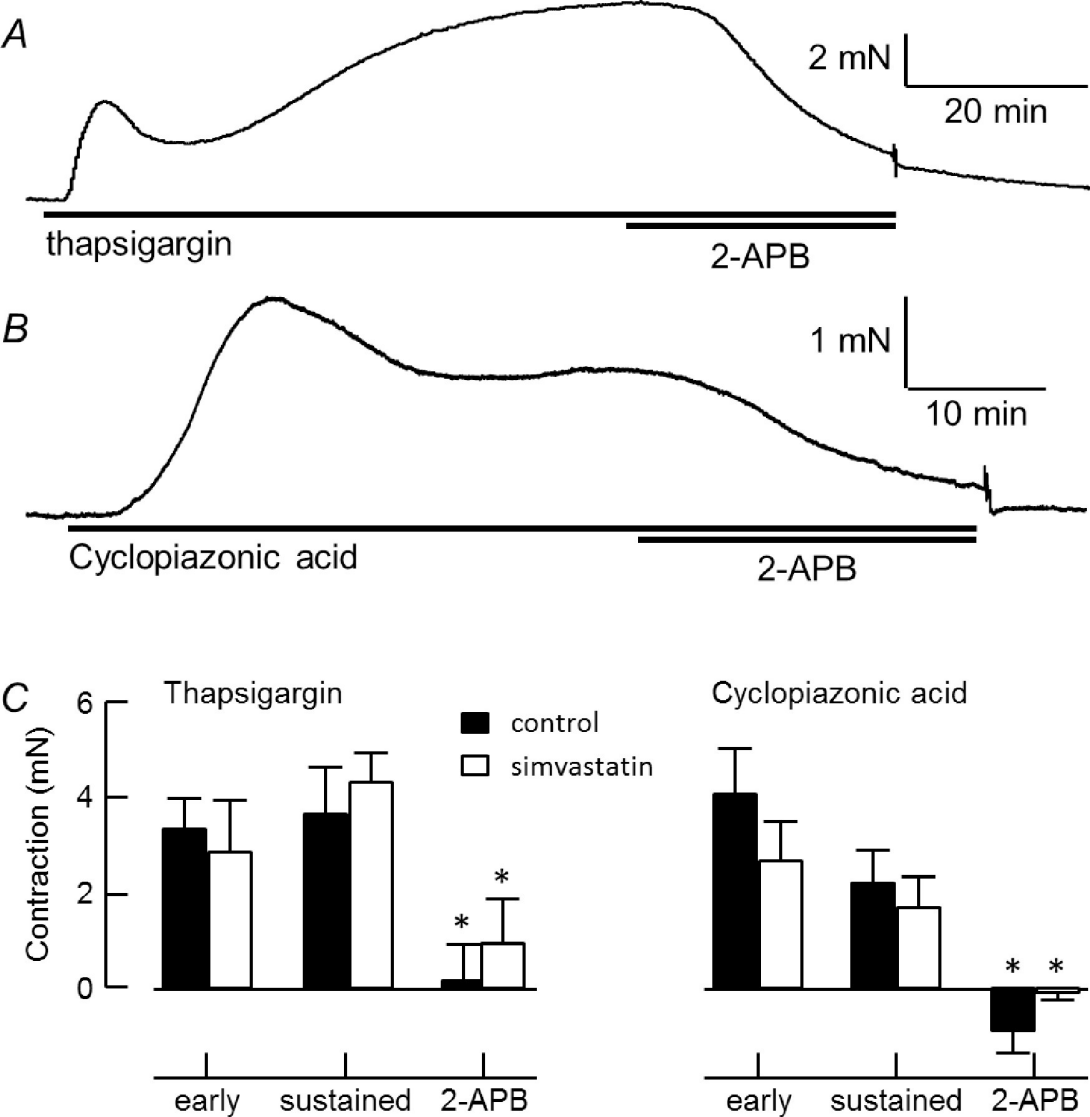
Simvastatin does not alter contraction evoked by SR Ca^2+^. Tension responses to 3 μM thapsigargin (*A*) or CPA (*B*), followed by 20 μM 2-APB. C, Early and sustained contraction of healthy arteries to thapsigargin or CPA (mean ± s.e.m., n = 7–9 rats), followed by tension remaining after application of 2-APB with and without exposure to 5 μM simvastatin. *differs significantly from peak and sustained responses (2-way RM ANOVA with Tukey’s multiple comparisons).

Inhibition of SERCA by 3 μM thapsigargin (Fig 6A) or 30 μM CPA (Fig 6B) evoked a rise in tension followed by decline to a lower level, which was usually sustained but sometimes rose again to a higher level before being maintained. When applied during the more sustained phase, the store-operated cation-channel blocker, 2-APB (20 μM), essentially abolished tension. The maximum tension developed by control arteries in response to thapsigargin or CPA was 4.7 ± 0.7 mN (n = 23) and 6.6 ± 1.2 mN (n = 12), respectively. Responses were reduced in arteries from MCT rats, where thapsigargin evoked 1.1 ± 0.3 mN (n = 17) of tension and CPA evoked 2.4 ± 0.3 mN (n = 23). As illustrated in Fig 6C, simvastatin did not significantly reduce responses to thapsigargin or CPA in control or MCT vessels. Neither the early nor the sustained phases of contraction were altered and 2-APB retained its ability to inhibit the sustained contraction.

### Effects of PAH and simvastatin on NO/cGMP-mediated vasodilation

**Fig 7:**
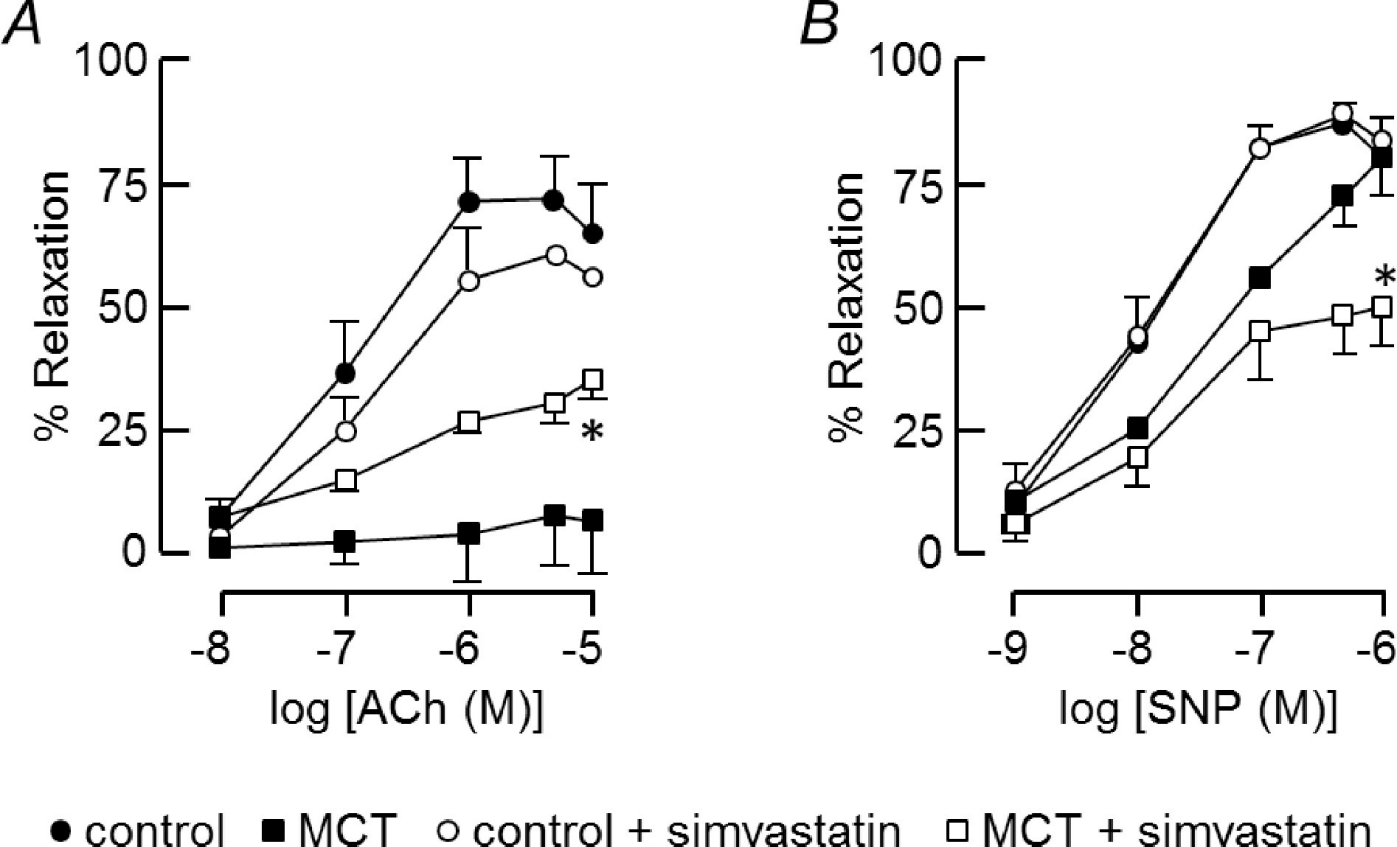
Simvastatin improves endothelial function in PAH. Concentration-response relationships for PA relaxation induced by ACh (A) and SNP (B) in arteries from control and MCT-treated rats, with and without simvastatin (5 μM) pre-treatment. Arteries were constricted with 30 nM U46629 until a stable plateau was reached then ACh (10 nM—10 μM) or SNP (1 nM—1 μM) was applied cumulatively. Relaxation was measured as percent of U46619-induced tone. Data represent mean ± s.e.m. of 6–9 animals. * differs significantly from the control response of MCT vessels at the same dilator concentration (unpaired t-test).

Acetylcholine (ACh) evoked concentration-dependent relaxation of U46619-constricted vessels from control animals (Fig 2*A*) but had little effect on MCT vessels (Fig 2*B*). Fig 7*A* compares ACh concentration-relaxation curves from control and MCT arteries, with and without simvastatin treatment. Control vessels relaxed with pEC_50_ = 6.9 ± 0.1 and E_max_ = 71 ± 11% (n = 6), values that were not significantly changed by simvastatin (pEC_50_ = 6.7 ± 0.1, E_max_ = 60 ± 11%, n = 6). In contrast, the maximum relaxation achieved by 10 μM ACh in MCT vessels was only 7 ± 10% (n = 9), but it reached 34 ± 4% (n = 6, P = 0.05, unpaired t-test) after simvastatin treatment.

Relaxation to SNP was also reduced in MCT vessels, but not restored by simvastatin (Fig 7*B*). SNP relaxed control arteries with pEC_50_ = 7.8 ± 0.2 and E_max_ = 87 ± 4% (n = 6) at ~1 μM, and following simvastatin treatment the values were unchanged (pEC_50_ = 7.96 ± 0.07, E_max_ = 89 ± 3%, n = 8). In MCT arteries, the SNP concentration-response curve was shifted to higher concentrations. As the maximum was not always clear, sensitivity to SNP was compared in control and MCT vessels using the SNP concentration that gave 50% reduction of the U46619-induced tone: 15 ± 0.4 nM (n = 6) in control vessels and 104 ± 2 nM (n = 8) in MCT vessels. Thus, there was a 7-fold loss of potency in vessels from MCT-treated rats (Mann-Whitney U Test). Simvastatin significantly inhibited responses to SNP in MCT vessels (two-way RM ANOVA), but only at SNP concentrations ≥100 nM.

## Discussion

Exposure of rat pulmonary arteries to simvastatin caused pronounced inhibition of vasoconstrictor responses to smooth muscle depolarisation (KCl), α_1_-adrenoceptor activation (phenylephrine), TP receptor activation (U46619) and eNOS inhibition (L-NAME). Simvastatin had the same effects on healthy and diseased vessels, except that it additionally reduced basal tension in diseased arteries. The inhibition of vasoconstrictor responses did not require an intact endothelium or functional eNOS, but resulted from direct interaction with smooth muscle cells. The ability of Y27632 to both mimic and prevent the effects of simvastatin on phenylephrine and U46619-induced contractions indicates the involvement of ROCK inhibition. This is supported by the loss of ROCK activity in simvastatin-treated arteries. The relative resistance of KCl-induced contraction to Y27632, despite inhibition by simvastatin, implies an additional action of simvastatin, independent of ROCK activity. The additional action could be inhibition of L-type Ca^2+^ channels, as suggested by simvastatin inhibition of Mn^2+^ quenching of fura-2 fluorescence. In contrast, simvastatin failed to inhibit contraction resulting from the blockade of SERCA (thapsigargin and CPA), which raises myocyte Ca^2+^ concentration *via* a mechanism that bypasses Ca^2+^ channels or receptor activation [32].

TP and α_1_-adrenergic receptors are G-protein coupled receptors (GPCRs). Both couple to G_q/11_ [33], which promotes the activation of RhoA [34,35] and downstream ROCK signalling. U46619 did not induce a detectable increase in the ROCK activity of isolated pulmonary arteries. ROCK is, however, expressed in all of the cells that comprise pulmonary arteries, including smooth muscle, endothelium and fibroblasts [36,37]. Thus changes in smooth muscle ROCK induced by TP receptor activation may not be large enough to detect above the background of activity from other cells. It proved impossible to isolate the smooth muscle and retain sufficient tissue for assaying ROCK activity. Although we tried to destroy the endothelium, its removal from all vessels could not be verified. Our result does not therefore conflict with the established ability of GPCRs to stimulate ROCK. Indeed, U46619 is known to stimulate the translocation of RhoA to the membrane and activate ROCK in cultured rat pulmonary artery smooth muscle cells [38]. By interfering with this pathway, simvastatin could inhibit RhoA/ROCK-dependent Ca^2+^-sensitisation of the contractile proteins, which maintains contraction during agonist stimulation [9]. Simvastatin must act on a pathway that is activated by GPCRs, as it failed to inhibit Ca^2+^-evoked contraction caused by inhibiting SERCA. Statins can disrupt GPCR signalling by interfering with the prenylation of Gγ subunits [39] and this could also contribute to the anti-contractile effect of simvastatin.

Ca^2+^-sensitisation is less important for contraction mediated by smooth muscle depolarisation. This was confirmed by the smaller effect of Y27632 on responses to KCl compared with receptor activation, in agreement with previous studies [10,11]. Additional mechanisms are therefore needed to explain why simvastatin reduced responses to KCl as effectively as it reduced GPCR-stimulated contraction. KCl contracts smooth muscle by depolarising the cell membrane and stimulating Ca^2+^ influx through voltage-gated, L-type Ca^2+^ channels. Consequently, the L-type Ca^2+^-channel blocker, nifedipine, almost abolished responses to KCl, while reducing phenylephrine and U46619 contraction by only 50% in control arteries. Simvastatin slowed the rate at which Mn^2+^ quenched fura-2 fluorescence in K^+^-depolarised pulmonary artery smooth muscle cells. As Mn^2+^ can only enter the cell to quench fluorescence when Ca^2+^ channels are open [30], this indicates that simvastatin inhibited Ca^2+^ influx. Thus, simvastatin most likely inhibited L-type Ca^2+^ channels as reported previously for basilar artery smooth muscle cells [12], cardiac myocytes [13] and pancreatic β-cells [40].

Simvastatin also inhibited contractile responses to L-NAME, which occur only in vessels with an intact endothelium [41–44]. L-NAME blocks the constitutive release of NO from the endothelium, which helps to maintain low resting tone by masking myogenic tone. The response amplitude was similar in healthy and diseased vessels, although eNOS inhibition has been reported to contract hypertensive arteries more effectively [41,42,44]. Both ROCK and Ca^2+^ entry through L-type channels have been shown to mediate contraction induced by L-NAME [41,45], so inhibition of these pathways could explain the susceptibility to simvastatin. In PAH, pulmonary arteries develop basal tension in the absence of stimulation, even when the endothelium is intact [42,44]; this is not seen in healthy arteries and is a recognised characteristic of PAH. The appearance of intrinsic tone in PAH is associated with progressive smooth muscle depolarisation [44] and can be reversed by nifedipine [41] and inhibitors of ROCK [46], implying the involvement of L-type Ca^2+^ channels and RhoA/ROCK-mediated Ca^2+^ sensitisation. Thus, nifedipine and Y27632 relaxed unstimulated arteries from MCT-treated animals, while having no effect on healthy vessels. The raised basal tone in hypertensive arteries was also suppressed by simvastatin, consistent with it inhibiting ROCK and Ca^2+^ channels.

We found no evidence that simvastatin suppressed contraction by stimulating AMPK and constitutive eNOS activity, as proposed to explain similar actions on mesenteric arteries [16]. While simvastatin could employ different mechanisms in different vascular beds, it seems unlikely. Although the earlier study found that dorsomorphin prevented simvastatin from inhibiting contraction, the concentration employed was10-fold higher than used here and 100-fold higher than required for 50% block of AMPK [27]. Dorsomorphin is non-specific above 1 μM and one of its many targets is the ROCK2 isoform [27,47]. Inhibition of ROCK activity may therefore account for the ability of dorsomorphin to prevent simvastatin from inhibiting mesenteric artery contraction.

Consistent with the findings of *in vivo* studies [], simvastatin exposure appeared to improve the dysfunctional endothelium that developed in PAH [17–20], although the effect was small. In this respect simvastatin affected diseased and healthy vessels differently. Healthy arteries showed no sign of enhanced relaxation either to ACh or the endothelium-independent dilator SNP. As simvastatin enhanced the relaxation of hypertensive arteries to ACh, but not SNP, this effect must be due to an action on the endothelium. During acute exposure to simvastatin, eNOS activity could be enhanced as a result of post-translational modification of the protein [48]. Longer exposure may be needed to increase eNOS expression and significantly restore endothelium-dependent dilation.

## Conclusions

The main effect of acute simvastatin exposure is to promote vasodilation through a direct interaction with smooth muscle. Simvastatin prevents vasoconstrictors from contracting smooth muscle and relaxes hypertensive arteries even in the absence of a constrictor stimulus. We propose that these effects are mediated by loss of ROCK-dependent Ca^2+^ sensitisation and inhibition of Ca^2+^ entry through L-type Ca^2+^ channels.

## Supporting information

S1 Fig Effects of Y27632 and nifedipine on contraction of endothelium-denuded pulmonary arteries. (PDF)

## Abbreviations

ACh: acetylcholine
AMPK: AMP-activated protein kinase
CPA: cyclopiazonic acid
EC_50_: molar concentration giving 50% of the maximum effect
E_max_: maximum effect
eNOS: endothelial nitric oxide synthase
GPCR: G protein-coupled receptor
HEPES: 4-(2-hydroxyethyl)piperazine-1-ethanesulphonic acid
L-NAME: N(G)-nitro-L-arginine methyl ester
LV+S: left ventricle plus septum
MCT: monocrotaline
PA: pulmonary artery
PAAT: pulmonary artery acceleration time
PAH: pulmonary arterial hypertension
PSS: physiological salt solution
ROCK: Rho-associated protein kinase
RV: right ventricle
S.E.M.: standard error of mean
SERCA: sarco/endoplasmic reticulum Ca^2+^ ATPase
SNP: sodium nitroprusside
TAPSE: tricuspid annular plane systolic excursion
U446619: 9,11-dideoxy-9a,11a-methanoepoxy prostaglandin F_2α_
Y27632: trans-4-[(1R)-1-aminoethyl]-N-4-pyridinylcyclohexanecarboxamide dihydrochloride
