# Supplementary material for: Simvastatin causes pulmonary artery relaxation by blocking smooth muscle ROCK and calcium channels: Evidence for an endothelium-independent mechanism
Source: PLoS One. 2019 Aug 1;14(8):e0220473. doi: 10.1371/journal.pone.0220473 (PMC6675113; doi:10.1371/journal.pone.0220473)
Supplement: S1 Fig — (PDF) [file pone.0220473.s001.pdf]

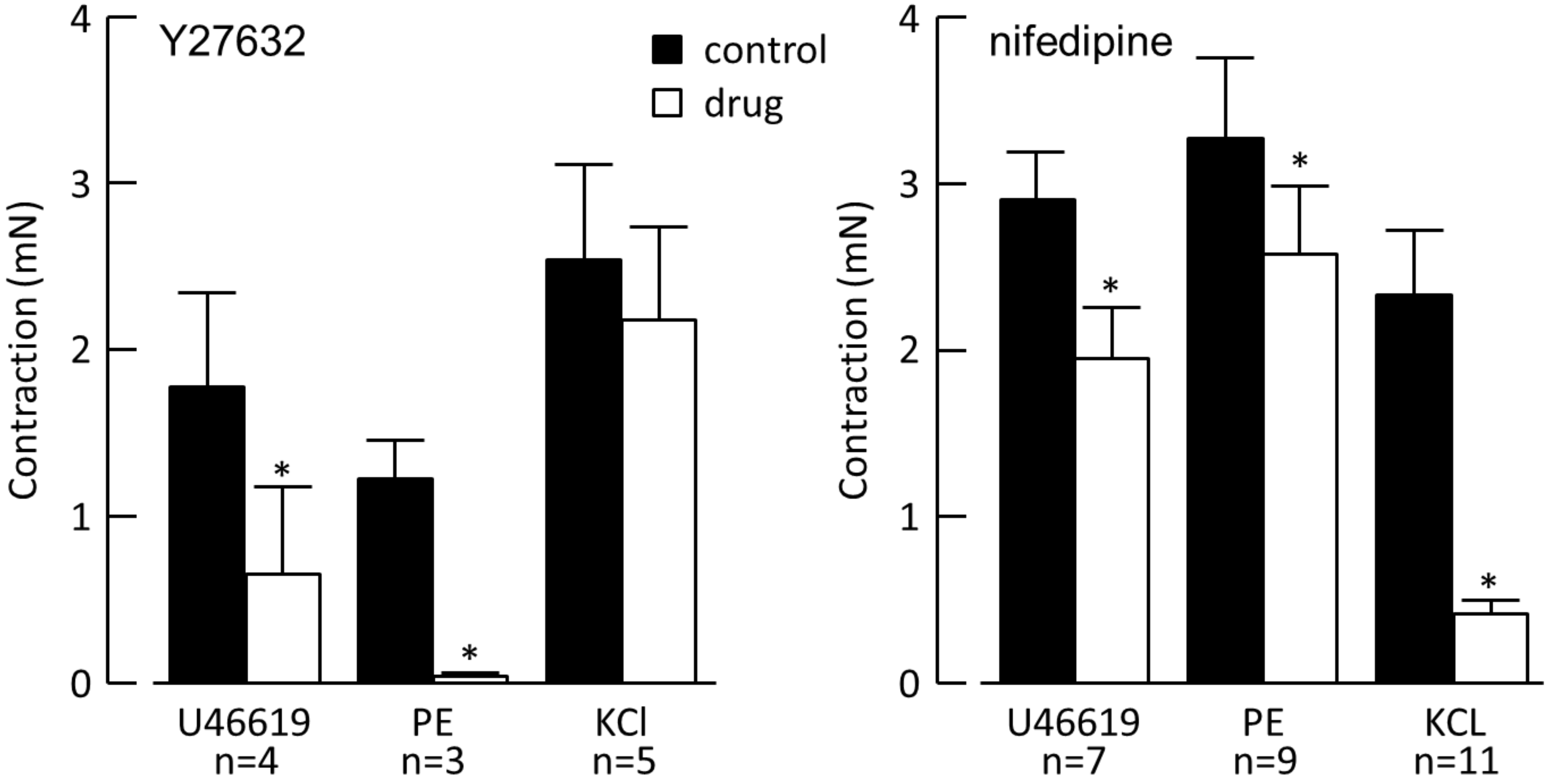

S1 Fig. Effects of Y27632 and nifedipine on contraction of endothelium-denuded pulmonary arteries. Contraction of healthy pulmonary arteries to 30nM U46619 , 5µM phenylephrine or 50mM KCl in control conditions and after 10 min exposure to 10µM Y27632 (left histogram) or 1µM nifedipine (right histogram). Distilled water was passed through the vessel lumen to remove endothelium. To ensure complete loss of endothelial function, vessels were also incubated with 200μM L-NAME and 10 µM indomethacin through the experiments. *differs significantly from control by 2-way RM ANOVA with Sidak's multiple comparisons test. Number of experiments indicated below bars.
